# Experimental α-particle radioimmunotherapy of breast cancer using ^227^Th-labeled p-benzyl-DOTA-trastuzumab

**DOI:** 10.1186/2191-219X-1-18

**Published:** 2011-08-24

**Authors:** Nasir Abbas, Helen Heyerdahl, Øyvind S Bruland, Jørgen Borrebæk, Jahn Nesland, Jostein Dahle

**Affiliations:** 1 Department of Radiation Biology, Institute for Cancer Research, Oslo University Hospital, Montebello, 0310 Oslo, Norway; 2 Faculty of Medicine, University of Oslo, P.O. Box 1074 Blindern, 0316 Oslo, Norway; 3 Department of Oncology, The Norwegian Radium Hospital, Oslo University Hospital, Montebello, 0310 Oslo, Norway; 4 Department of Pathology, Oslo University Hospital, Montebello, 0310 Oslo, Norway; 5 Algeta ASA, Kjelsås, 0411 Oslo, Norway

**Keywords:** alpha radiation, radioimmunotherapy, SKBR-3, trastuzumab, thorium-227

## Abstract

**Background:**

The aim of the present study was to explore the biodistribution, normal tissue toxicity, and therapeutic efficacy of the internalizing low-dose rate alpha-particle-emitting radioimmunoconjugate ^227^Th-trastuzumab in mice with HER2-expressing breast cancer xenografts.

**Methods:**

Biodistribution of ^227^Th-trastuzumab and ^227^Th-rituximab in nude mice bearing SKBR-3 xenografts were determined at different time points after injection. Tumor growth was measured after administration of ^227^Th-trastuzumab, ^227^Th-rituximab, cold trastuzumab, and saline. The toxicity of ^227^Th-trastuzumab was evaluated by measurements of body weight, blood cell, and clinical chemistry parameters, as well as histological examination of tissue specimens.

**Results:**

The tumor uptake reached peak levels of 34% ID/g (4.6 kBq/g) 3 days after injection of 400 kBq/kg of ^227^Th-trastuzumab. The absorbed radiation dose to tumor was 2.9 Gy, while it was 2.4 Gy to femur due to uptake of the daughter nuclide ^223^Ra in bone; the latter already explored in clinical phases I and II trials without serious toxicity. A significant dose-dependent antitumor effect was observed for dosages of 200, 400, and 600 kBq/kg of ^227^Th-trastuzumab but no effect of 400 and 600 kBq/kg ^227^Th-rituximab (non-tumor binding). No serious delayed bone marrow or normal organ toxicity was observed, but there was a statistical significant reduction in blood cell parameters for the highest-dose group of ^227^Th-trastuzumab treatment.

**Conclusion:**

Internalizing ^227^Th-trastuzumab therapy was well tolerated and resulted in a dose-dependent inhibition of breast cancer xenograft growth. These results warrant further preclinical studies aiming at a clinical trial in breast cancer patients with metastases to bone.

## Background

Metastatic breast cancer patients have poor prognosis despite recent therapeutic advances [1]. The human epidermal growth factor receptor-2 (HER-2/neu) is a transmembrane receptor tyrosine kinase that is over-expressed in 25% to 30% of metastatic breast cancers and associated with more aggressive disease [2]. Trastuzumab (Herceptin^®^) is a humanized monoclonal antibody (mAb) directed against this antigen and shows clinical activity in women both with HER2/neu-overexpressing primary and metastatic breast cancer [3].

Tumor cell-targeted alpha emitters have the potential to improve therapy of hematological malignancies and micrometastatic disease. Alpha particles have a short path length (50 to 80 μm) and high linear energy transfer (LET approximately 100 keV/μm) and, thus, deliver a high amount of DNA-damaging energy to cells in close vicinity of their decay. However, no alpha-emitting radioimmunoconjugate (RIC) has reached phase III clinical trial yet due to poor physical or chemical characteristics, supply limitations, and high production costs for the most promising alpha emitters [4]. Recently, we have suggested ^227^Th as a novel radionuclide for alpha-particle radioimmunotherapy (RIT), as this radionuclide can be produced in clinically relevant amounts from β-decay of the long-term generator ^227^Ac [5,6]. ^227^Ac can be produced by thermal neutron irradiation of ^226^Ra in a nuclear reactor. The yield of ^227^Ac after purification is relatively high and ^226^Ra is highly available, making the process cost efficient. ^227^Ac has a half-life of 21.8 years and thus, would serve as a generator nuclide for ^227^Th production for decades [7].

Thorium-227 decays via its alpha- and beta-emitting daughters ^223^Ra, ^219^Rn, ^215^Po, ^211^Pb, ^211^Bi, and ^207^Tl to stable 207 Pb. The long half-life of ^227^Th (*T*_1/2 _= 18.7 days) permits the tumor targeting and normal tissue clearance of a ^227^Th-labeled RIC to occur before larger amounts of the daughter nuclide ^223^Ra is generated. Upon decay, ^223^Ra will detach from the antibody. Importantly, clinical trials have not shown worrisome toxicity of ^223^Ra injected as a therapy for prostate cancer bone metastases [8,9]. Previously, we have shown that ^227^Th conjugated to the monoclonal antibody rituximab was effective in treatment of mice with lymphoma xenografts and had a relatively low normal tissue toxicity [7,10,11].

The conjugation of trastuzumab with different alpha-particle-emitting radionuclides, i.e., ^211^At, ^225^Ac, and ^213^Bi, has already been investigated by other groups [12-16]. The purpose of the present study was to determine the biodistribution, therapeutic effect, and toxicity of the low-dose rate alpha-particle-emitting RIC ^227^Th-trastuzumab on HER2-expressing SKBR-3 xenografts. *In vitro *experiments have shown internalization of the ^227^Th-trastuzumab/HER2 complex, retention of ^227^Th, and a high toxic effect against single tumor cells [17]. The increased cytotoxic effect created by alpha particles may offer the opportunity to both improve the overall response rate of the trastuzumab treatment and also to treat patients with a lower HER2 expression.

## Material and methods

### Production of ^227^Th and radiolabeling of monoclonal antibodies

^227^Ac was produced through thermal neutron irradiation of ^226^Ra followed by β^- ^decay of ^227^Ra (*T*_1/2 _= 42.2 min) to ^227^Ac [18]. ^227^Th was selectively retained from a ^227^Ac decay mixture in 7 M HNO_3 _by anion exchange chromatography [19].

Radiolabeling of trastuzumab (Herceptin^®^, Hoffmann-La Roche, Basel, Switzerland) and rituximab (MabThera, Hoffmann-La Roche) with ^227^Th was performed at Algeta ASA, Oslo, Norway. The antibodies were conjugated with p-SCN-Bn-DOTA at pH 9 (sodium borate buffer) at 37°C over night. The number of DOTA molecules per antibody was approximately four as determined by LC/MS analysis. The conjugate was purified with a spin filter (Amicon, Millipore, USA) using 0.9% NaCl as running buffer removing daughter nuclides and non-chelated ^227^Th. The purified antibody was distributed to microcentrifuge tubes (1 mg/tube) and freeze dried to keep a larger batch under stable conditions over a long period of time. The freeze-dried conjugate was dissolved in sodium acetate buffer pH 5.5 and added about 4 MBq of newly purified ^227^Th in 0.01 M HCl. The reaction was done over night at 42°C in a thermomixer (Eppendorf, Hamburg, Germany). The chelate was purified on a NAP5 column (GE Healthcare, Little Chalfont, UK) using PBS as running buffer. The specific activity was 1000-1600 kBq/mg with regard to ^227^Th.

### Immunoreactivity

The immunoreactive fraction (IRF) of the radioimmunoconjugate ^227^Th-trastuzumab was estimated by measuring the cell bound activity in a one point assay. SKOV-3 cells (2 × 10^7 ^cells/ml) in 200 μl PBS were used. Four million SKOV-3 cells in one vial of cells were blocked by incubating with 150 μg/ml cold trastuzumab for 15 min at 37°C. The cells in another vial were not blocked. About 500 cpm of ^227^Th-trastuzumab was added to each vial and the cells were incubated for 2 h before washing and measurement of radioactivity with an automated gamma counter (Wizard, Packard Instrument Co., Downers Grove, IL, USA). IRF was 70% to 90%.

### Animals

All procedures and experiments involving animals in this study were approved by the National Animal Research Authority and carried out according to the European Convention for the Protection of Vertebrates used for Scientific Purposes. The animals were maintained under pathogen-free conditions. Food and water were supplied *ad libitum*. Eight to 12 weeks old, institutionally bred female Balb/C nu/nu (NCR) mice, with an average weight of 20 to 27 g at the start of study, were used. Mice were anesthetized with subcutaneous injection of 0.05 ml Zoletil^® ^mix (Virbac, Carros Cedex, France) before HER-2-positive breast cancer (SKBR-3) tumor fragments from xenografted animals (1 × 1 × 1 mm) were implanted subcutaneously. The xenografted tumor line originated from HER-2-positive breast cancer (SKBR-3) cells from American Type Culture Collection (Manassas, VA). Mice with growing tumors of diameters between 4 and 8 mm were included in the experiments. Mice were killed by cervical dislocation.

### Biodistribution of ^227^Th-labeled antibodies

The conjugates ^227^Th-trastuzumab and ^227^Th-rituximab were administered by tail vein injection of 100 μl (15 kBq) solution to each animal. For each conjugate and time point, a total of four to six animals were autopsied. Tumor and organs were measured for radioactivity content and weighed. Samples of the injectates (10%) were used as references in the measurement procedure.

Thorium-227 and ^223^Ra were measured using a solid-state photon well detector (GCW6021, Canberra, Meridan, CT, USA) coupled to a digital gamma ray spectrometer and analyzed using the computer software Apex™ version 1 (Canberra). For ^227^Th, the 236 keV (abundance 17.6%) and 256 keV (abundance 9.5%) γ-ray lines were used and for ^223^Ra the 154 keV (abundance 5.7%), 269 keV (abundance 13.9%), 324 keV (abundance 4%), and 338 keV (abundance 2.8%) γ-ray lines were used.

### Calculation of absorbed dose

The total number of disintegrations, i.e., the cumulated activity, in various tissues from the time of injection of the preparation until no activity was left in the body was estimated by calculation of the area under the activity concentration versus time curves (AUC). For ^227^Th-labeled antibodies, the absorbed radiation doses were calculated assuming dose contributions coming only from α-particle emissions with a mean α-energy (E_α_) of 5.9 MeV for ^227^Th and 26.4 MeV for ^223^Ra with its daughters in equilibrium, and that there was a 100% absorption of the absorbed dose from the α-particle within a tissue, i.e., absorbed fraction equal to unity (*ø *= 1). For α-particle radiation uniform distribution of radionuclides in the various tissues as well as no cross irradiation was assumed. Thus, the total absorbed dose to each organ was estimated by: Dose = AUC_0_^∞ ^· E_α _(^227^Th) + AUC_0_^∞ ^· E_α _(^223^Ra + daughters). Also for blood, the absorbed dose was calculated assuming 100% absorption of the α-particles, i.e., *ø *= 1. This was obviously a simplification since in the capillaries there will probably be escape of α-particles beyond the blood.

### Therapeutic studies

Mice were injected with a single dose of NaCl (control; *n *= 10), 20 μg (*n *= 5), 100 μg (*n *= 6), or 250 μg (*n *= 5) of cold trastuzumab; 200 kBq/kg (*n *= 10), 400 kBq/kg (*n *= 11), and 600 kBq/kg (*n *= 12) of ^227^Th-trastuzumab; and 400 kBq/kg (*n *= 9) and 600 kBq/kg (*n *= 10) ^227^Th-rituximab in 100 μl solution. Tumor growth and mouse weight were assessed three times a week in the first week before injection and the 3 weeks after injection; thereafter, weight, growth, and survival were assessed twice a week. Caliper measurements of perpendicular tumor diameters were used to estimate tumor volume by assuming ellipsoid shape. Mice with tumor diameter larger than 20 mm were killed. Mantley Cox log rank test was used to test for significant differences in surviving fraction of mice, which is defined as the fraction of mice that did not have to be sacrificed due to tumor diameter above 20 mm.

### Evaluation of toxicity

Toxicity was evaluated in all treatment groups except ^227^Th-rituximab. Approximately 100 to 200 μl of blood was collected from the vena saphena lateralis in 500 μl EDTA-coated tubes (Microtainer K2E tubes, Becton, Dickinson, NJ, USA) for blood cell counting. Blood samples were taken before and at 3, 6, and 8 to 10 weeks after start of the study. For control, a group of ten mice without tumor was injected with NaCl and sampled at the same time points for blood cell count. While for clinical chemistry data, the samples from this control group were taken after 8 weeks. Blood cells were counted in an automatic blood counter (Scil Vet ABC, Horiba ABX, Montpellier, France). In addition, when mice were sacrificed due to tumor size or weight loss, blood samples were collected by heart puncture into EDTA-coated tubes and also lithium heparin-coated tubes (Microtainer LH tubes, Becton, Dickinson) for analysis of clinical chemistry parameters. Clinical chemistry strips were used to assess the serum aspartate aminotransferase (AST), alanine aminotransferase (ALT), alkaline phosphatase (ALP), and urea level (Reflotron, Roche Diagnostics GmbH, Mannheim, Germany). Full blood samples (30 μl) were analyzed by a clinical chemistry analyzer (Reflovet, Roche Diagnostics).

At the end of the study, the lung, heart, kidney, spleen, small intestine, large intestine, liver, femur, skull, and tumor were fixed with formalin, cut in 5-μm slices, stained with hematoxylin and eosin, and analyzed by a pathologist to detect any pathological changes. Slides from cold trastuzumab and ^227^Th-trastuzumab groups were compared to the slides of control groups.

### Autoradiography

Mice bearing tumor xenografts were injected with 15 kBq of ^227^Th-trastuzumab, corresponding to approximately 600 kBq/kg. Four animals were sacrificed by cervical dislocation 4 and 8 days after injection. Tumors were removed and immediately frozen in liquid nitrogen. Tissue sections of thickness 5 μm were used for exposure of Kodak Biomax MR-1 single-sided emulsion or Kodak Medical General Purpose Blue x-ray film (Eastman Kodak Company, Rochester, NY, USA). Films were exposed for 6 to 11 days at -80°C prior to development. Film patterns were compared to hematoxylin and eosin (H/E)-stained tissue sections.

## Results

### Biodistribution and dosimetry of ^227^Th -trastuzumab and ^227^Th-rituximab

**Figure 1:**
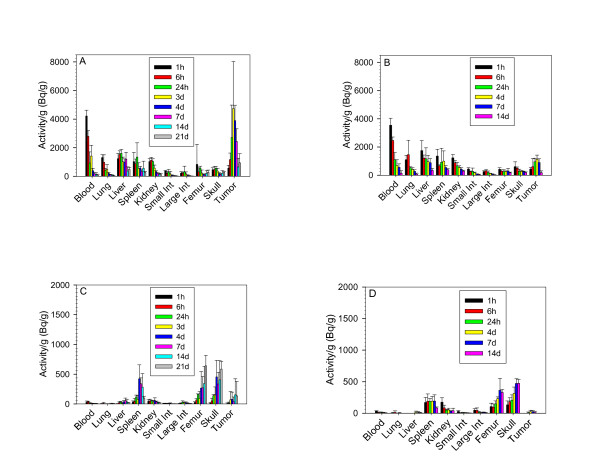
**Biodistribution of ^227^Th-conjugates and ^223^Ra in mice with SKBR-3 xenografts**. Biodistribution profile of ^227^Th-trastuzumab (**a**) and daughter nuclide ^223 ^Ra (**c**) after administration of ^227^Th-trastuzumab, and biodistribution of ^227^Th-rituximab (**b**) and daughter nuclide ^223 ^Ra (**d**) after administration ^227^Th-rituximab, in mice bearing SKBR-3 xenografts. The measured ^227^Th activities were normalized to an injection of 400 kBq/kg bodyweight. Values are mean ± SD. *N *= 6 for each time point except at day 3, where *N *= 5.

The *in vivo *biodistribution profiles of ^227^Th-trastuzumab, ^227^Th-rituximab, and the daughter nuclide ^223^Ra in nude mice with SKBR-3 xenografts at different time points after administration are shown in Figure 1. The maximum uptake of ^227^Th-trastuzumab in tumor (4.6 kBq/g) occurred 3 days after injection (Figure 1a). There was a large difference between the amount of activity in tumor and in normal organs for ^227^Th-trastuzumab. The uptake of non-tumor binding ^227^Th-rituximab (Figure 1b) in tumor was significantly lower than the uptake of ^227^Th-trastuzumab (Figure 1a). The ^227^Th daughter nuclide ^223^Ra was mainly localized to bone (femur and skull) but there were also some retention of ^223^Ra in spleen, kidneys, and in tumor (Figure 1c, d).

**Figure 2:**
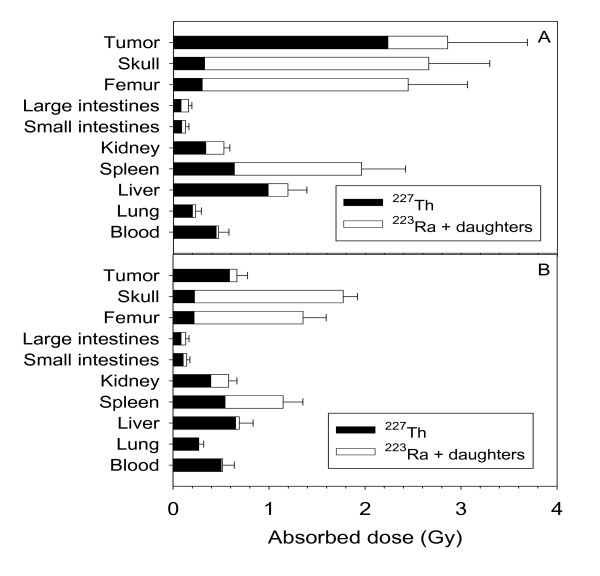
**Absorbed radiation doses to normal tissues and tumor xenografts**. Absorbed radiation dose in tumor and normal organs of mice injected with ^227^Th-trastuzumab (**a**) or ^227^Th-rituximab (**b**). Cumulated activities were calculated from biodistribution curves and multiplied with the mean energy of α-particles from ^227^Th, ^223^Ra, and daughters. Biodistribution data of ^227^Th-trastuzumab and ^227 ^Th-rituximab were normalized to 400 kBq/kg bodyweight.

The absorbed radiation dose in tumor was 2.9 ± 0.8 Gy for ^227^Th-trastuzumab (Figure 2a) and 0.7 ± 0.1 Gy for ^227^Th-rituximab (Figure 2b); both normalized to injections of 400 kBq/kg. Radiation doses were less than 2 Gy for all organs for both RICs, except for femur (2.4 ± 0.6 Gy) and skull (2.7 ± 0.6 Gy) in mice treated with ^227^Th-trastuzumab.

### Therapeutic efficacy

**Figure 3:**
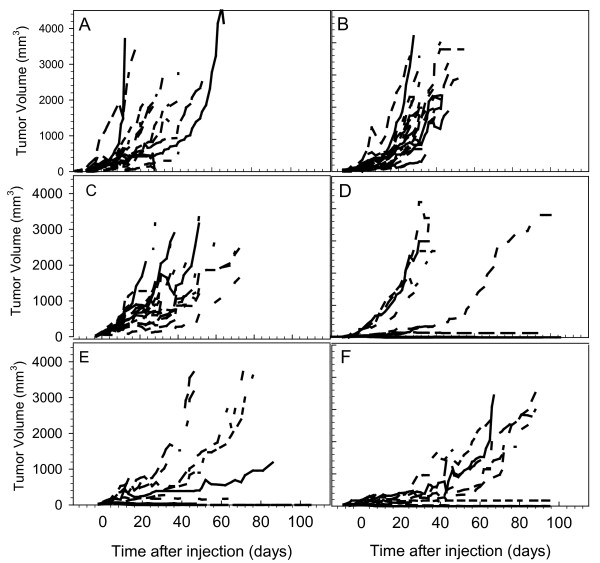
**Effects of ^227^Th-based RIT on growth of individual SKBR-3 tumor xenografts**. Individual tumor growth after treatment with NaCl (**a**); 20, 100, and 250 μg cold trastuzumab (**b**); ^227^Th-rituximab at dosage of 400 and 600 kBq/kg (**c**); 200 kBq/kg (**d**); 400 kBq/kg (**e**) and 600 kBq/kg (**f**) of ^227^Th-trastuzumab. *N *= 9 to 19.

Growth of SKBR-3 tumor xenografts in mice treated with alpha-particle-emitting ^227^Th-trastuzumab was compared with cold trastuzumab, non-tumor binding ^227^Th-rituximab, as well as saline (controls; Figure 3). There was a large variability in tumor growth within treatment groups. Table 1 shows growth delays calculated from average tumor growth curves. The mean tumor growth in mice treated with cold trastuzumab (20, 100, and 250 μg/mice or approximately 0.8, 4, and 10 mg/kg body weight) or 400 and 600 kBq/kg ^227^Th-rituximab was similar to the growth of the untreated controls. The dosage groups for cold trastuzumab and for ^227^Th-rituximab in Table 1 and Figure 3b, c were pooled since there was no difference between them. For 200 and 400 kBq/kg ^227^Th-trastuzumab, some of the tumors responded well to the treatment, while others did not (Figure 3d, e). The average delays to grow to a normalized tumor volume of 500 mm^3 ^were 7 and 23 days, respectively (Table 1). For 600 kBq/kg ^227^Th-trastuzumab, all tumors responded to the treatment (Figure 3f) and the average growth delay to reach a tumor volume of 500 mm^3 ^was 45 days (Table 1).

**Table 1:** Growth inhibition for tumor volume of 500 and 1,000 mm^3 ^after treatment

Treatment	Dosage	**500 mm**^**3**^		**1,000 mm**^**3**^	
		**Days**^**a**^	Growth delay^b^	Days	Growth delay
NaCl		15 ± 7	0 ± 10	25 ± 7	0 ± 11
Trastuzumab (pooled)	20 - 250 μg	15 ± 7	0 ± 11	23 ± 8	-2 ± 12
^227^Th-rituximab	400 and 600 kBq/kg	15 ± 8	-3 ± 11	27 ± 10	2 ± 11
^227^Th-trastuzumab	200 kBq/kg	22 ± 8	7 ± 11	32 ± 8	7 ± 12
^227^Th-trastuzumab	400 kBq/kg	38 ± 8	23 ± 10	70 ± 10	45 ± 12
^227^Th-trastuzumab	600 kBq/kg	60 ± 7	45 ± 7	90 ± 10	65 ± 7

^a^The number of days to reach the chosen tumor volume. ^b^Growth delay = days (treatment) - days (NaCl).

**Figure 4:**
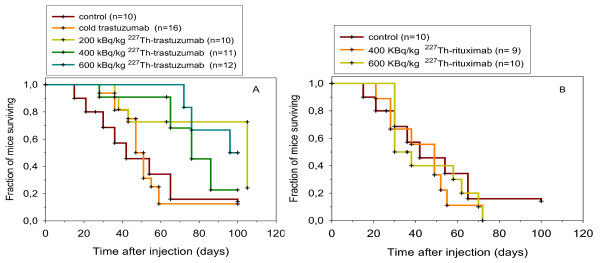
**Effects of ^227^Th-based RIT on survival of mice with SKBR-3 tumor xenografts**. Survival of mice after intravenous injection of NaCl, 20,100, and 250 μg cold trastuzumab, and 200, 400, and 600 kBq/kg ^227^Th-trastuzumab (**a**), or 400 and 600 kBq/kg ^227^Th-rituximab (**b**).

The surviving fraction of the different dosages of ^227^Th-trastuzumab was not significantly different from each other (*p *> 0.05), but there was a significant difference in survival between the ^227^Th-trastuzumab treatment groups and control groups (NaCl and trastuzumab; *p *< 0.001) (Figure 4a). Mean and median survival times were significantly different for mice in the dosage groups 400 and 600 kBq/kg ^227^Th-trastuzumab as compared to mice in the NaCl (control) group (*p *< 0.05; Table 2). None of the dosages of cold trastuzumab had an effect on survival (*p *= 0.40). Hence, the data were pooled into one group. The survival of mice treated with non-tumor-binding ^227^Th-rituximab was not significantly different from the survival of the control group (*p *> 0. 6; Figure 4b). In addition, no significant differences (*p *> 0.05) in mean and median survival times between control and ^227^Th-rituximab treatment groups were observed (Table 2).

**Table 2:** Mean and median survival times for all treatment groups

Treatment	Dosage	Mean ± standard error	Median ± standard error	Number of mice
NaCl		52 ± 10	42 ± 13	10
Trastuzumab (Pooled)	20 - 250 μg	54 ± 5	47 ± 2	16
^227^Th-rituximab	400 kBq/kg	44 ± 5	49 ± 8	9
^227^Th- rituximab	600 kBq/kg	47 ± 5	36 ± 3	10
^227^Th-trastuzumab	200 kBq/kg	39 ± 2	38 ± 2	10
^227^Th-trastuzumab	400 kBq/kg	87 ± 7*	63 ± 3*	11
^227^Th-trastuzumab	600 kBq/kg	95 ± 3*	96 ± 3*	12

*Significant difference between control and therapy.

### Toxicity of ^227^Th-trastuzumab

**Figure 5:**
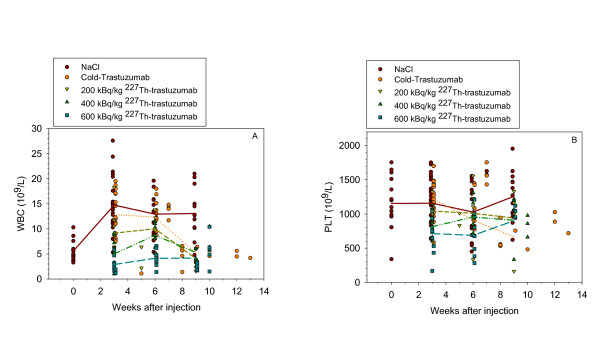
**Blood cell counts after ^227^Th-trastuzumab therapy**. Assessment of bone marrow toxicity estimated by white blood cell counts (**a**) and platelet counts (**b**) as a function of time after administration of NaCl, cold trastuzumab, and 200, 400, and 600 kBq/kg of ^227^Th-trastuzumab. Line graphs shows means of each treatment group.

**Figure 6:**
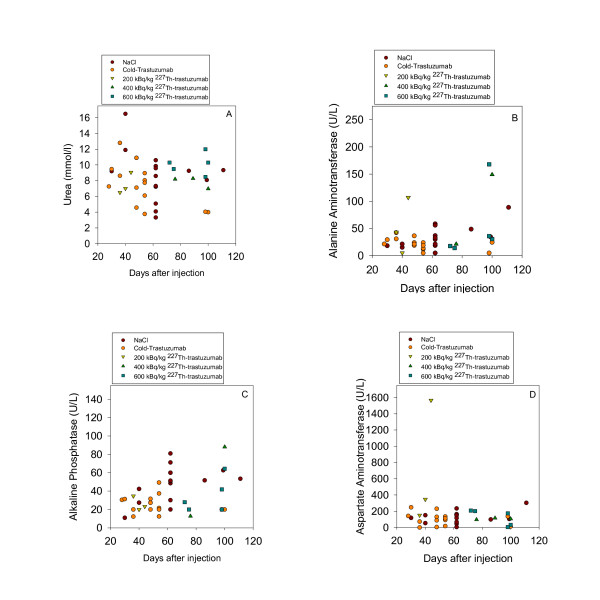
**Assessment of liver and kidney functions after ^227^Th-trastuzumab therapy**. Measurement of urea (**a**), ALT (**b**), ALP (**c**), and AST (**d**) concentration in blood of mice with time after administration of NaCl, cold trastuzumab, 200, 400, and 600 kBq/kg of ^227^Th-trastuzumab.

White blood cell (WBC), platelet cell (PLT) counts, and clinical chemistry parameters of control mice and mice treated with ^227^Th-trastuzumab are shown in Figures 5 and 6. Figure 5a, b shows WBC and PLT counts of individual mice as well as mean values at 0, 3, 6, and 9 weeks time points from each treatment groups. In the control groups mice without tumor was also included in order to get measurements at longer follow-up. The WBC count was significantly lower in the control (NaCl) group at time 0 as compared to 3 weeks after injection. WBC decreased significantly for treatment with 400 kBq/kg (*p *< 0.001, *t *test) and 600 kBq/kg (*p *< 0.001, *t *test) of ^227^Th-trastuzumab as compared to WBC in the cold trastuzumab group and control mice after 3 weeks (Figure 5a) but not as compared with the 0 time point. After 6 weeks, only the 600 kBq/kg ^227^Th-trastuzumab group was significantly different from control (*p *= 0.008, *t *test).

No significant difference in PLT count was found for the 200 kBq/kg ^227^Th-trastuzumab treatment group when compared to control at any time point (Figure 5b). The PLT count was significantly lower for the 400 kBq/kg (*p *= 0.017, *t *test) and 600 kBq/kg (p = 0.003, *t *test) ^227^Th-trastuzumab treatments as compared to control after 3 weeks. At 6 weeks, the PLT counts had recovered. However, at 9 weeks, the PLT count was significantly lower than the control for the 400 kBq/kg ^227^Th-trastuzumab group (*p *= 0.038, *t *test) and for the cold trastuzumab group (*p *< 0.001, *t *test) as compared to control mice.

Urea, AST, ALT, and ALP levels in blood from control mice were compared with blood from mice treated with cold trastuzumab, 200, 400, and 600 kBq/kg of ^227^Th-trastuzumab (Figure 6a, b, c, d). Urea levels were within the normal range and were not significantly different from control. One mouse in each RIT group and one control mouse showed high ALT levels, i.e., above normal range. Another mouse treated with 200 kBq/kg ^227^Th-trastuzumab group had a very high AST levels as compared to mice in all other treatment groups. Large variations in ALP levels were observed among all treatment groups but were within the normal range. Therapy related pathological changes were not observed in any organ upon histological examination.

**Figure 7:**
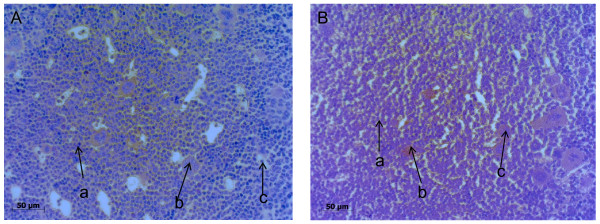
**Histological examination of bone marrow after ^227^Th-trastuzumab therapy**. Histological microscopy images of bone marrow in femur of mice after administration of NaCl (**a**) or 600 kBq/kg ^227^Th-trastuzumab (**b**) showing islands of haemopoetic cells composed of blood cells in various stages of maturation (arrow a), a great population of nucleated blood cells (arrow b), and blood vessels (arrow c).

Figure 7 shows no morphological differences in normal bone marrow for a mouse treated with NaCl (Figure 7a) and a mouse treated with 600 kBq/kg of ^227^Th-trastuzumab up to 72 days (Figure 7b). Body weights of animals were measured throughout the study but no significant differences between the treatment groups were observed (data not shown).

### Autoradiography

**Figure 8:**
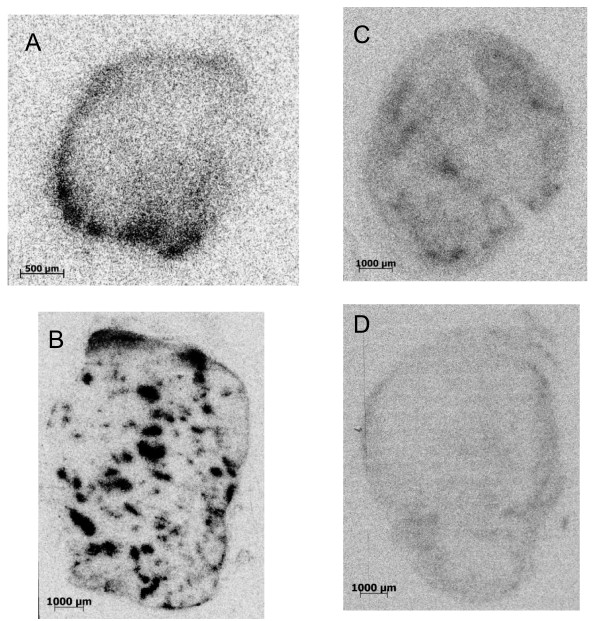
**Autoradiography images after ^227^Th-trastuzumab therapy**. Autoradiography images of the radioactivity distribution in 5-μm-thick frozen tissue sections from four different SKBR-3 human tumor xenografts in athymic nude mice following injection of 600 kBq/kg of ^227^Th-trastuzumab. Tumors in mages (**a**) and (**b**) were resected 4 days post injection, while (**c**) and (**d**) were removed 8 days post injection. *N *= 4.

Autoradiography of SKBR-3 tumor xenografts showed that the distribution patterns of radioactivity after injection of 600 kBq/kg of ^227^Th-trastuzumab were inhomogeneous (Figure 8). The smallest of the tumors analyzed showed highest concentration of radioactivity present as a rim corresponding to areas with viable tumor tissue close to the well perfused connective tissue capsule surrounding the tumor (Figure 8a). The tumor in Figure 8b had localized hotspots. On the corresponding H/E-stained tissue section, the hotspots with high ^227^Th-trastuzumab uptake matched areas with high density of blood vessels and/or large blood vessels, with areas of necrotic tissue and loosely bound cells in between. A similar correspondence was also seen in tissue sections taken at later time points (Figures 8c, d).

## Discussion

The present study of alpha-particle-emitting ^227^Th-trastuzumab showed a significant dose-dependent inhibition of tumor growth of human SKBR-3 breast cancer xenografts in mice, leading to long-term survival with low toxicity.

In RIT with ^227^Th the distribution of free daughter nuclides also has to be considered, as the daughter nuclide ^223^Ra detaches from the DOTA-trastuzumab construct upon alpha-particle emission from ^227^Th. The biodistribution study showed that ^223^Ra re-localized to bone and to spleen. It should be noticed that the 18.7-day half-life of ^227^Th allows for excretion of a large fraction of ^227^Th-trastuzumab before ^223^Ra is formed. The uptake in spleen was probably related to mouse-specific calcification of the spleen [20]. Radium-223 has a half-life of 11.4 days and is excreted from the blood via the intestines with a major part of the ^223^Ra ending up in the hydroxyapatite of bone [8,20,21]. The half-lives of the ^223^Ra-daughters are in the millisecond to minute range. They are therefore likely to contribute mainly to the absorbed radiation dose in the vicinity of the site of ^223^Ra decay. Thus, as shown in Figure 2 the absorbed doses to bone were comparable to the doses in tumor.

Microautoradiography studies of ^227^Th-rituximab have shown that there probably is a contribution to the bone marrow absorbed dose from ^223^Ra and daughters on the bone surface [11]. One could suspect that localization in bone would give a high contribution to bone marrow toxicity, but clinical studies of ^223^Ra have shown that it is well tolerated by breast and prostate cancer patients [8], with data from repeated dosing suggesting no more damage on red bone marrow compared to placebo [9]. This lack of toxicity is probably due to the short path length of alpha emission, as previous data have shown that the beta-emitter strontium-89 is strikingly more toxic, although presumably localizing in an identical way in bone tissue [20]. Therefore, we suggest that localization of small amounts of ^223^Ra in bone tissue would be acceptable. Furthermore, because of the long half-life of ^227^Th and internalization of HER-2 antigen after binding to ^227^Th- trastuzumab complex much of the ^227^Th will be excreted or internalized before ^223^Ra is formed and thereby reducing relocalization of ^223^Ra to bone. We also suggest that an optimized chelator will reduce the small amounts of free ^227^Th, indicated by the present biodistribution data.

No severe bone marrow toxicity was observed in this study even when therapeutically effective amounts were administered. A dosage of 600 kBq/kg of ^227^Th-rituximab is equal to an absorbed radiation dose in tumor of around 1 Gy. One could expect a small therapeutic effect of this dose since there was a significant therapeutic effect of 200 kBq/kg (1.45 Gy) of ^227^Th-trastuzumab. However, there was no therapeutic effect of even the highest dosage of ^227^Th-rituximab, showing that the antibody has to bind to the cells to get the emitted alpha particles close enough to the tumor cell nucleus. This is in analogy with the lack of bone marrow toxicity, discussed above, i.e., the low bone marrow toxicity might be due to the lack of binding of ^227^Th-trastuzumab or ^223^Ra to bone marrow cells.

In the present study, the tumor volumes were 8 to 16 times larger than the size of micrometastases (< 2 mm in diameter) in breast cancer patients. However, in a previous study we treated single SKBR-3 cells and achieved up to two log reduction in clonogenic survival and growth inhibition [17]. Therefore, one relevant clinical setting for ^227^Th-trastuzumab might be adjuvant treatment of breast cancer patients with micrometastases. Due to the ^223^Ra (daughter) affinity to bone, patients with a high risk of developing bone metastasis might be an intriguing application [22,23].

There was a dosage-dependent increase in tumor growth inhibition but not for survival. This may be related to individual differences in tumor vascularization and the presence of necrosis. In the 200 kBq/kg group we observed a variable therapeutic effect, while in the 400 and 600 kBq/kg groups we got a more prominent and similar therapeutic effect.

Radiolabeled antibody therapy for solid tumor has been less successful as compared to hematological tumors. The reasons are that the solid tumors are generally less sensitive to radiation and are more difficult to target due to macromolecule transport barriers, e.g., vascular supply limitation, high interstitial pressure, and vascular permeability limitation. Targeted delivery of high LET α-particles after administration of ^227^Th-trastuzumab may not be the only reason behind the successful growth inhibition of SKBR-3 tumor xenografts. The autoradiography images indicated that ^227^Th-trastuzumab in some tumors, were located close to the tumor vasculature. Targeting the tumor vasculature or vasculature near the tumor cells with α-emitting radionuclides has previously been shown to yield a therapeutic effect on solid tumors [24,25].

The tumors treated in the present study were much larger than the range of alpha particles. However, the autoradiogaphy images indicated hot spots of ^227^Th-trastuzumab activity in perfused areas within the tumor xenografts, which might result in destruction of the blood vessels and eradication of tumors due to lack of nutrients. Furthermore, there was also some retention of free ^223^Ra in tumor. This is a small ion with several α-emitting daughter radionuclides that might surmount the macromolecular transport barriers of solid tumors and result in high LET α-irradiation of tumor cells not reached by the larger molecule ^227^Th-trastuzumab. Thus, the antitumor effect might have been a combined effect of tumor cell kill by both ^227^Th-trastuzumab, ^223^Ra, and daughters and destruction of the blood vessels that delivers nutrients and oxygen to the tumor cells.

At two samplings, the dosage-dependent decrease in the WBC count was significantly lower for mice in one or both of the two highest dosages groups compared to control mice; both for the 400 and 600 kBq/kg ^227^Th-trastuzumab groups at 3 weeks after injection, and for the 600 kBq/kg^227^Th-trastuzumab at 6-week time points. However, the blood values were within the normal physiological range for nude mice for all dosages of ^227^Th-trastuzumab. Furthermore, the most striking change in the WBC count is the increase for the control group from 0 to 3, 6, and 9 weeks. If the WBC count at 3, 6, and 9 weeks are compared with the WBC count of the control at 0 weeks there is no significant difference. The reason for this increase is unknown, but it might be related to an undetected infection in one cage of the control mice. Therefore, we conclude that the ^227^Th-trastuzumab treatment had no pathological effect on the WBC count.

There was a dosage-dependent decrease in PLT count at 3 weeks after injection, but the PLT count had recovered after 6 weeks. At the 9-week time point, the PLT count was significantly lower than the control for the 400 kBq/kg ^227^Th-trastuzumab group and the cold trastuzumab group. However, this decrease was probably related to a combination of biological variation and the low number of mice in these two groups (5 and 1, respectively). It should also be pointed out that control mice without tumor xenografts were used in order to get blood samples for the controls at the later time points.

Liver enzymes and urea levels in the blood did not show any dose-dependent changes following injection of ^227^Th-trastuzumab with levels in the highest dosage group similar to that of control. Other treatment groups showed random increase or decrease of some enzymes when compared with control. This may be related to one mouse within each group with very high value of the parameter in question. Since there were no dose-dependent changes and since there were no significant changes between the control and the 600 kBq/kg group for any parameters, these changes might be due to other factors than the ^227^Th-trastuzumab treatment.

In conclusion, ^227^Th-trastuzumab inhibits growth of breast cancer xenografts in a dose-dependent manner. Possibly due to the longer half-life, single dosing was efficacious; not excluding that improved efficacy might be obtained with multiple doses, as has been shown clinically with a more short-lived alpha emitter [9]. The limited toxicity of the treatment was mainly related to reversible bone marrow depression. Further preclinical studies of ^227^Th-trastuzumab involving mice with breast cancer micrometastases and, if possible, metastasis to bone are warranted.

## Competing interests

JB is an employee of Algeta ASA which owns the patents for using ^227^Th in radioimmunotherapy. JD and OSB own a small amount of shares in Algeta ASA.

## Authors' contributions

NA designed and performed the invivo studies and carried out interpretation and analysis of data including dosimetry calculation and writing of manuscript. JB performed radiolabeling and contributed in manuscript writing. JN and NA carried out histopathological studies of slides. HH carried out the autoradiography studies including interpretation and analysis of these data, and contributed to performing experiments and writing of parts of the manuscript. ØSB contributed to the study design, interpretation and analyses of data as well as writing of the manuscript. JD contributed to the study design, interpretation and analyses of data, writing the manuscript as well as performing experiments and dosimetry calculations. All authors read and approved the final manuscript.
